# A Comparative Analysis of Structural Brain MRI in the Diagnosis of Alzheimer’s Disease

**DOI:** 10.3233/BEN-2009-0225

**Published:** 2009-10-21

**Authors:** Jason Appel, Elizabeth Potter, Qian Shen, Gustavo Pantol, Maria T. Greig, David Loewenstein, Ranjan Duara

**Affiliations:** ^1^Mount Sinai Medical CenterWien CenterMiami BeachFLUSA; ^2^Department of Biomedical EngineeringUniversity of MiamiMiamiFLUSA; ^3^Departments of MedicineUniversity of MiamiNeurology and Psychiatry and Behavioral SciencesMiller School of MedicineUniversity of MiamiMiamiFLUSA; ^4^Department of RadiologyMount Sinai Medical CenterMiami BeachFLUSA; ^5^Johnnie B. ByrdSr. Alzheimer’s Center & Research InstituteTampaFLUSA; ^6^Department of NeurologyUniversity of South FloridaTampaFLUSA

## Abstract

Dementia is a debilitating and life-altering disease which leads to both memory impairment and decline of normal executive functioning. While causes of dementia are numerous and varied, the leading cause among patients 60 years and older is Alzheimer’s disease. The gold standard for Alzheimer’s diagnosis remains histological identification of amyloid plaques and neurofibrillary tangles within the medial temporal lobe, more specifically the entorhinal cortex and hippocampus. Although no definitive cure for Alzheimer's disease currently exists, there are treatments targeted at preserving cognition and memory while delaying continued loss of function. Alzheimer's disease exists along a spectrum of cognitive decline and is often preceded by Mild Cognitive Impairment (MCI). Patients with MCI demonstrate memory loss and cognitive impairment while still continuing normal activities of daily living, and are considered to be at increased risk for developing Alzheimer's Dementia. Identifying patients with prodromal states of Alzheimer's dementia such as MCI may allow initiation of appropriate treatment planning and delay of cognitive decline. Therefore, the need for a non-invasive early biomarker for the detection of Alzheimer's disease has never been greater. Multiple neuroimaging methods utilizing visual rating scales, volumetric measurements, and automated methods have been developed to identify, quantify, and track anatomic sequelae of Alzheimer’s Disease.

## 1. Introduction

Alzheimer's disease is a gradually progressive illness that is often preceded by a prodromal condition known as Mild Cognitive Impairment (MCI), in which memory loss and other cognitive functions are impaired, even though the patient may continue to perform normal activities of daily living adequately [26]. Patients with MCI are considered to be at significantly increased risk for developing AD within three to five years [27]. Identifying patients with MCI or prodromal states of Alzheimer's dementia becomes critical as initiation of pharmacological and immunotherapy treatment may be most efficacious during the earliest stages of AD. Although currently no decisive cure for AD exists, therapies are being developed for preservation of cognition and memory while delaying continued loss of function.

For elderly patients it would be optimal to develop early, non-invasive biomarkers for detecting or predicting onset of AD. Although a definitive diagnosis of AD requires post-mortem histological confirmation by identifying amyloid neuritic plaques and neurofibrillary tangles within the neocortex, the earliest pathological changes of this disease occur in medial temporal lobe structures, specifically the entorhinal cortex (ERC) and hippocampus (HPC) [5]. Atrophy of the HPC, documented by antemortem magnetic resonance

ISSN 0953-4180/09/$17.00 © 2009 – IOS Press and the authors. All rights reserved images (MRIs) has been shown to correlate with severity of AD-related neuropathological changes [15,18]. On average, subjects diagnosed with mild cognitive impairment (MCI) show more atrophy of medial temporal lobe structures than cognitively normal subjects of the same age [12,27], and MRI studies of MCI subjects indicate that atrophy in the ERC and HPC predicts conversion to AD [17]. The forgoing suggests that neuroimaging may be an important tool in the diagnosis of prodromal AD.

## 2. Visual rating methods assessing brain MRIs

Neuroimaging methods that utilize visual rating scales, automated software, and volumetric measurements have been developed to identify, quantify, and track medial temporal lobe atrophy (MTA). Although volumetric analysis software is readily available, in a clinical setting diagnosis generally relies on the visual assessment of two dimensional MRI scans. Major reasons for this include the following: (1) Volumetric analysis of brain scans requires pre-processing of data in order to meet requirements of the volumetric program; this typically requires specific expertise and additional time; (2) about 5 to 10% of scans that can be analyzed by semiquantitative visual rating methods are not suitable for volumetric studies, as they include movement or other artifacts, or were created using inadequate acquisition methodologies; (3) many volumetric methods measure pre-programmed and fixed regions, whereas measuring volumes of specific regions that may be affected during early stages of AD may require the manual tracing of brain structures by a trained neuroanatomist, or development of customized computer programs; (4) volumetric methods cannot assess various anatomical and qualitative pathological changes that occur during the course of AD, such as gray-white matter distinction, which is an independent measure of pathological change.

Scheltens and colleagues [30] have developed a visual rating method to perform semi-quantitative measurements of atrophy severity, for the entire medial temporal region on each side of the brain, using coronal slices of brain MRI scans. This group demonstrated that visual rating of MTA on MRIs is an efficient method, which does not require extensive personnel training, expensive equipment, or the preparation of imaging data that is associated with volumetric analysis [10,30,35, 40]. The visual rating method was shown to be capable of distinguishing early AD subjects from those with no cognitive impairment (NCI), and predicting which subjects would convert from MCI to AD [40]. Inter- and intra-rater reliability for assessing atrophy in the entire medial temporal region proved to be adequate, although rating of individual medial temporal structures was found to be less than optimal [30]. Wahlund et al. [40] compared qualitative visual rating of MTA, using a 0 to 4 scale, with "no atrophy" rated as "0," and "severe atrophy" rated as "4", to volumetric analysis of the hippocampus. They showed that visual ratings of MTA demonstrated greater overall sensitivity (95%), specificity (98%), and correct classification (96%), than volumetric analysis (93% correct classification), in separating AD from non-dementia patients [40]. This visual rating method also performed better than volumetric analysis in discriminating AD from other dementias [40]. Moreover, Visser et al. [39] demonstrated that Scheltens' visual rating method for MTA was a better predictor of delayed recall performance, in comparison to volumetric analysis of the parahippocampal gyrus.

The scope and utility of Scheltens' system was expanded by Urs et al. [35] and Duara et al. [10], to provide reliable visual ratings of individual medial temporal lobe (MTL) structures, i.e., hippocampus (HPC), entorhinal cortex (ERC) and perirhinal cortex (PRC). Visual ratings were performed on 1.2 mm to 1.5 mm thick coronal slices positioned perpendicular to the AC-PC line and intersecting the mammillary bodies. To enable high reliability, a Visual Rating System (VRS) program was developed, which includes reference images to promote standardization among users, improves accuracy in atrophy ratings, and increases inter-rater and intra-rater reliability [35]. In a study of 261 community-dwelling subjects, aged 65 and older, a minority of whom were enrolled at memory disorder clinics in Miami and Tampa, VRS was used to assess MTA on structural brain MRIs. The subjects were diagnosed to have no cognitive impairment (NCI), non-amnestic MCI (rarely a precursor to probable AD), amnestic MCI (often a precursor to probable AD), and probable AD [10]. The optimal predictive power for VRS was obtained by averaging the scores of three medial temporal structures on each side. This provided a sensitivity of 85% and specificity of 82%, for diagnosing Probable AD, and a sensitivity of 80% and specificity of 82%, for diagnosing amnestic MCI [10]. MTA scores also distinguished Probable AD and aMCI subjects, from naMCI and NCI subjects, and predicted transition from NCI to MCI, and from MCI to probable AD.

Accuracy of the VRS in measuring MTA was compared to a volumetric method (IBASPM, www.

thomaskoenig.ch/Lester/ibaspm.htm) in the diagnosis of AD. In a preliminary study, participants in the Florida Alzheimer Disease Research Center were diagnosed as cognitively normal (NCI; $n = 32$), amnestic MCI (aMCI; $n = 45$), or with Dementia (DEM; $n = 30$) [31]. Area under the receiver operating curve (aROC) for the left hemisphere in DEM versus NCI groups was 0.80 for volumetry, and 0.83 for VRS ($p =$ NS). Similar results were obtained in the right hemisphere for the same groups. For aMCI versus NCI, aROC for the right hemisphere was 0.73 for VRS, and 0.61 for volumetry ($p = 0.05$). Correlations of right and left MTA with scores on an episodic memory test ($r = 0.55$ and $r = 0.55$) were slightly higher with VRS, in comparison to volumetric measures ($r = 0.51$ and $r = 0.44$) [31].

## 3. Volumetric analysis of brain MRIs

Methodology for whole brain and regional volumetric analysis is widely available. One such method, the Individual Brain Atlas and Statistical Parametric Mapping (IBASPM) (www.thomaskoenig.ch/Lester/ibaspm.htm) is an extension of SPM5 (Welcome Department of Cognitive Neurology, London, UK), and is a freely available MRI software package executed in MATLAB (Natick, MA). In IBAPSM, the volume of brain structures is calculated after normalization or spatial transformation to templates obtained from the Montreal Neurological Institute (MNI) website (http://www.bic.mni.mcgill.ca/brainweb/). The scans are segmented into three types of tissue in each hemisphere: gray matter, white matter, and cerebral spinal fluid. An individual brain atlas for each subject is created with the transformation matrix obtained from the normalization step, and anatomical automatic labeling (AAL) ( http://www.cyceron.fr/freeware/) to specify 116 structures. The structures measured include the hippocampus, parahippocampal gyrus, and amygdala in the medial temporal lobe, where atrophy has been strongly correlated to neurodegenerative changes during AD. In different studies, manual outlining of serial coronal images to capture the entire volume of a structure has provided volumetric data for the hippocampus, putamen and thalamus, entorhinal cortex, perirhinal cortex, and posterior parahippocampal cortex. In these studies, a trained operator is required to manually segment the regions of interest, rendering this method expensive and time-consuming. Excellent discrimination between cognitively normal, MCI and AD subjects is possible using these volumetric methods [2, 8,9,12,29,33]. An alternative efficient, albeit relatively expensive program for automatic volumetric analysis of brain MRIs has been developed by NeuroQuant (CorTech Labs, La Jolla, CA).

Entorhinal cortex and hippocampal volume loss on MRI scans are highly correlated with progression rate of MCI to AD. Smaller hippocampal and entorhinal cortical size on MRIs have been related to memory impairment in normal aging and subjects at risk for future AD [17]. The presence of MCI or mild dementia, versus normal cognition has been associated specifically with atrophy of the left hippocampus, parahippocampal gyrus, and amygdala [4,19]. Even among subjects who are cognitively normal, those with subjective symptoms of cognitive impairment were found to have lower hippocampal volume [36]. The histopathological correlate of imaging findings appears to be accumulation of neurofibrillary tangles, neuritic plaques, and loss of neurons and dendritic arbor in the trans-entorhinal and hippocampal cortex [4,15,18]. Presence of medial temporal atrophy is not specific for AD, as fronto-temporal lobar dementia (FTLD), vascular dementia, and hippocampal sclerosis may demonstrate brain atrophy in these regions.

A conceptual point to be made is what additional variables shown to be associated with increased risk, or increased probability of progression from normal cognition to MCI, include age, memory scores, hypertension, and ApoE 4 genotype. Some of these are true risk factors, in the sense of participating in a causal pathway, while others appear to be epiphenomena, or effects of the disease process. Association with an elevated probability does not necessarily translate into improving sensitivity, specificity, or predictive value of diagnosis. Conceptually, certain factors are less suited to being considered as diagnostic.

## 4. Support vectors machines for automated classification of brain MRIs

Support vector machines (SVMs) are software programs designed to automate the classification of data. Computer-based, fully automated support vector machines (SVMs) have been designed to evaluate structural brain MRIs in the diagnosis of AD [24], normal aging, and frontotemporal lobar degeneration [22]. Such SVMs employ a set of related supervised learning tools for the regression analysis and classification of MRI data; SVMs "maximize" the separation between two data groups, and classify novel data into either group [22]. Specifically, SVMs use a complex algorithm on well-characterized data to discriminate the differences between groups. For example, during the first step the SVM can "learn" and document the differences in digitalized brain MRIs of two diagnostic groups, e.g., AD and healthy controls. Then, the SVM applies its "knowledge" to assign new cases to diagnosis groups. A caveat is that separation must be based on disease-related changes [22]. Kloppel and colleagues [22] reported high sensitivity (95–100%) and specificity (86–95%) generated by SVM, in comparison to radiologists' reports in the diagnosis and differentiation of AD and MCI from normal subjects.

The SVM may be a useful adjuvant tool; it is quite accurate, requires minimal supervision, and a small amount of user training. However, while SVMs can discriminate between normal aging and AD, or MCI groups, it is not certain whether SVMs work well to differentiate patients with common metabolic or structural abnormalities, from AD or MCI patients. Severe involutional changes, hydrocephalus, brain infarcts, post-surgical changes, hemorrhages, and neoplastic disorders are only a few examples that can easily produce alteration in the brain anatomy, and in these cases, SVM would likely not yield an accurate result. On the other hand, such concerns would not challenge a trained radiologist who would spend very little time using a visual rating method to diagnose MCI or AD.

Li and colleagues [23] have built an effective SVM classifier trained to detect changes in hippocampal subregions that are significant in discriminating AD patients and healthy control subjects. In this study, accuracy in classifying subjects based on SVM assessment of the bilateral hippocampal subregions was greater than 80%. Magnin and colleagues [25] have developed an SVM classifier of whole-brain anatomy that is recorded on MRIs, to discriminate AD patients and elderly control subjects. This SVM was designed to perform a histogram analysis of voxel intensities for each region of interest (ROIs) on the brain MRI. Next, a parameter that characterizes relative weight of gray matter, compared to white matter and cerebrospinal fluid is extracted. Then, the SVM sums extracted parameters for all ROIs of the whole brain MRI and classifies the subject using a complex mathematical algorithm. With this SVM, Magnin and colleagues obtained 94.5% correct classification of AD and control subjects, with 96.6% specificity, and 91.5% sensitivity. Kloppel and colleagues [22] utilized SVM to classify mild AD, FTLD, and control subjects. This SVM was used to evaluate volumes of gray matter for the whole brain, or for the anteromedial lobe volume only, as the input feature of the SVM classifier. In this study, up to 96% of pathologically verified AD patients were correctly classified using the whole brain images.

## 5. White matter hyperintensities (WMHs), MTA and AD

White matter hyperintensities (WMHs) appear as bright foci on T2-weighted MRI scans, [19] and may be observed in elderly subjects who are cognitively normal, diagnosed with MCI, or a variety of dementias including AD [12] [13,14,28]. Etiology of WMHs is commonly ascribed to normal aging and cerebrovascular disease, even among subjects diagnosed with probable AD [14,34]. Yet growing evidence indicates neurodegenerative processes, such as gliosis, microglial infiltration, inflammation, and amyloid angiopathy can generate WMHs [3,7,11,16,41]. Diverse etiologies and underlying neuropathologies [16], as well as coexisting and mutually synergistic effects of vascular and degenerative disease [6,21,37] may confound efforts to characterize a relationship between WMHs, MTA, and AD. The distinction between degenerative and vascular disease in the brain has become increasingly blurred at a pathological and clinical level, so that WMHs in the brain should be generally considered a manifestations of both disease processes [38].

In a study of more than 3000 subjects, Longstreth et al. [24] reported significant relationships between WMHs, demographic variables, cardiovascular risk factors, and found that cognitive performance was closely associated with severity of WMHs. On the other hand, although Staekenborg and colleagues [33] documented a clear association of MTA with WMHs on visually rated brain MRIs of AD patients, this group reported "no differences in behavior or psychological symptoms according to MTA or WMHs." Nevertheless, most studies have demonstrated a relationship between WMHs and current cognitive status and/or risk for future cognitive decline. Recently, Van Straaten and colleagues [37] used Scheltens' standardized visual rating scale to assess brain MRIs of 182 subjects in a longitudinal study, and found that periventricular white matter hyperintensities were related to increased risk of AD within three years. Convincingly, in a longitudinal study of cognitively normal adults who were assessed with volumetric analysis of brain MRIs, Rosano and colleagues [28] found that MTA and WMHs were "independently and significantly associated" with a greater

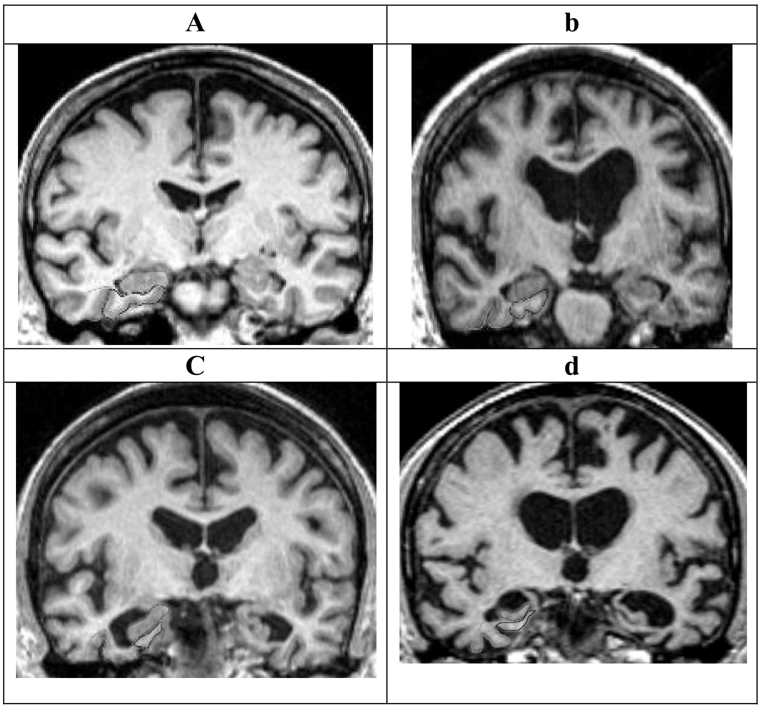

Fig. 1. Examples of reference images for the MB slice. The three regions of interest are outlined in the right hemisphere in color (HPC = red; ERC = blue; PRC = green). In Fig. 1 all three structures have no atrophy (Score = 0) in both hemispheres; In Fig. 2 all three structures have minimal (Score = 1) to mild (Score = 2) atrophy; in Fig. 3 all three structures have moderate (Score = 3) atrophy, although the left HPC is more atrophic than the right; in Fig. 4 all structures have severe atrophy (Score = 4), with the exception of the right PRC, which has moderate atrophy. (Note: a useful marker distinguishing mild from moderate atrophy is the loss of gray-white matter distinction in the ERC and the PRC).

risk to develop AD within 5 years. Furthermore, in this study it was found that coincidence of both MTA and WMHs "increased risk of developing AD by sevenfold" [28].

Appel and colleagues [1] rated MTA and white matter hyperintensities (WMHs) in 192 elderly subjects that were clinically and neuropsychologically diagnosed as cognitively normal, non-amnestic MCI (naM-CI), amnestic MCI (aMCI), and probable AD. Scores of WMH and MTA were greater in subjects with probable AD, relative to NCI and na-MCI subjects [1]. Overall correct classification rates of Probable AD versus NCI, using VRS MTA scores was 81.8%, improving to 86.5% when combined with WMH scores. WMHs were significantly related to MTA scores, but not to the cardiovascular risk factor scores, suggesting that in this cohort WMHs on MRI scans were primarily associated with neurodegenerative disease [1].

In summary, it appears likely that MRI measures will have continuing utility for research studies in dementia and MCI syndromes. Volumetric measures that are currently most frequently employed for research studies, have particular utility for determining changes in brain morphology longitudinally. Support vector machines may have particular applications for analyzing volumetric data. However, volumetric analysis has inadequacies in measuring small irregular structures such as the ERC [35], which reduces its sensitivity and specificity for detecting morphological changes that occur early in the course of Alzheimer's disease. Furthermore, volumetry requires high image quality and well-trained personnel to prepare and analyze the images, and it is not unusual for scans to be found unsuitable for volumetric analysis because of movement or other artifacts. Semiquantitative measures using visual rating for measuring medial temporal atrophy may be a more accurate method than volumetry, especially for cross-sectional studies, when the goal is to validate the diagnosis of AD. Semi-quantititative visual rating, especially using VRS, can detect atrophy in small irregular structures such as the ERC. At the same time the criteria for image acquisition are much less stringent; very few MRI scans are found unsuitable for visual rating because of artifacts, poor image quality or acquisition technique. Visual rating of MRI images can be incorporated into the routine radiological assessment of

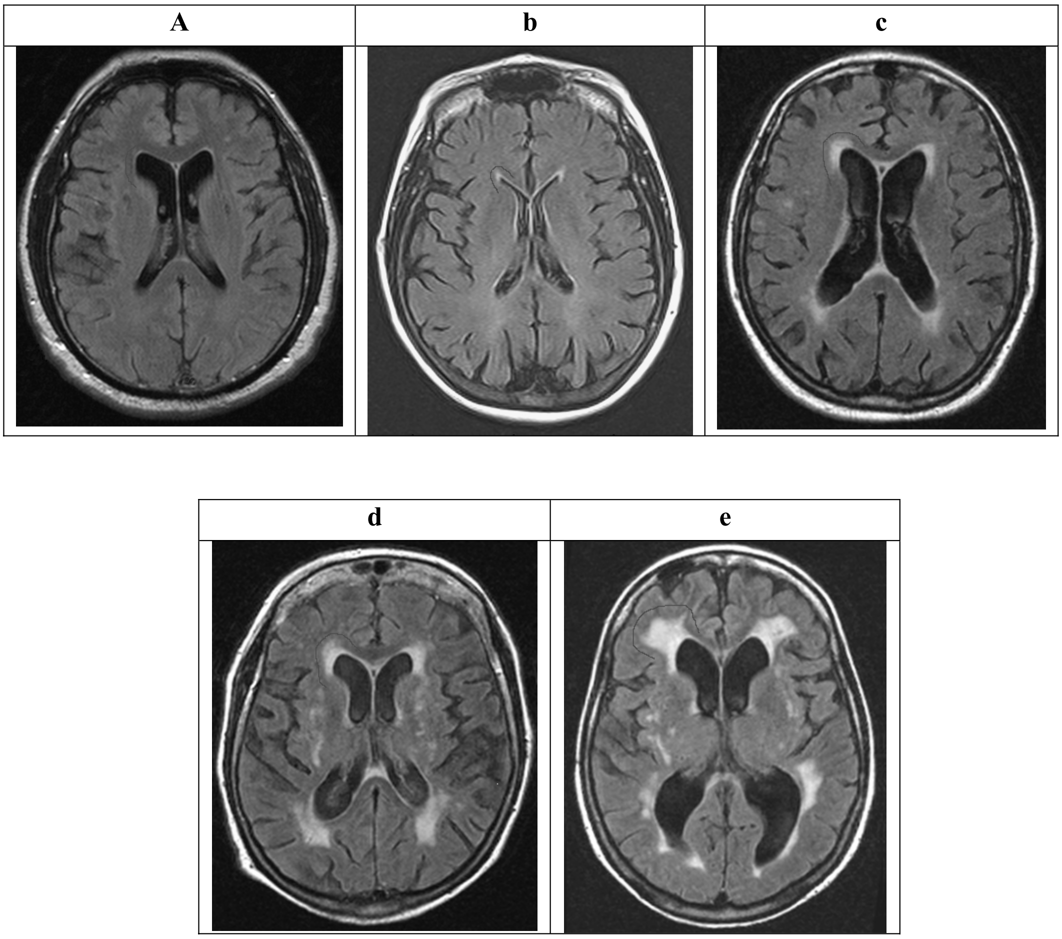

Fig. 2. White matter hyperintensities (WMHs) and Visual Ratings. Figures show examples of WMHs in the bilateral Periventricular (PVM) Frontal regions on a FLAIR axial brain MRI that would be visually rated at a level of 0 (a), 1 (b), 2 (c), 3 (d), and 4 (e). Examples of WMHs in the bilateral PVM Posterior regions are shown in Fig. 2 (d), (e); these WMHs would be rated at a level of 3, excepting the right posterior WMH region in (e), which would be rated at a level of 2.

MRI brain scans, adding very little additional reading time for a trained radiologist or technician. It is likely that semiquantitative visual rating will become a convenient and cost-efficient method to aid in the diagnosis of Prodromal AD and Probable AD.

## Acknowledgment

This research was supported by 1P50AG025711-01 from the National Institute of Aging and by a grant from the Byrd Alzheimer Center and Research Institute, Tampa, Florida.
